# Origin and Future of Plasmonic Optical Tweezers

**DOI:** 10.3390/nano5021048

**Published:** 2015-06-12

**Authors:** Jer-Shing Huang, Ya-Tang Yang

**Affiliations:** 1 Department of Chemistry, National Tsing Hua University, Hsinchu 30013, Taiwan; E-Mail: jshuang@mx.nthu.edu.tw; 2 Center for Nanotechnology, Materials Sciences, and Microsystems, National Tsing Hua University, Hsinchu 30013, Taiwan; 3 Frontier Research Center on Fundamental and Applied Science of Matters, National Tsing Hua University, Hsinchu 30013, Taiwan; 4 Department of Electrical Engineering, National Tsing Hua University, Hsinchu 30013, Taiwan

**Keywords:** optical tweezers, optical trapping, plasmonics, Brownian motion, nanomanipulation, surface plasmon, nanoplasmonics, near-field optics

## Abstract

Plasmonic optical tweezers can overcome the diffraction limits of conventional optical tweezers and enable the trapping of nanoscale objects. Extension of the trapping and manipulation of nanoscale objects with nanometer position precision opens up unprecedented opportunities for applications in the fields of biology, chemistry and statistical and atomic physics. Potential applications include direct molecular manipulation, lab-on-a-chip applications for viruses and vesicles and the study of nanoscale transport. This paper reviews the recent research progress and development bottlenecks and provides an overview of possible future directions in this field.

## 1. Introduction

Light or electromagnetic fields have linear momentum, and the transfer of this momentum to an object can result in the production of radiation forces. This fundamental fact was the driving force that inspired Arthur Ashkin to invent optical manipulation techniques on the microscopic scale or, equivalently, the field of optical tweezers [1,2,3,4,5,6,7,8,9,10]. For conventional optical tweezers based on the far-field technique with optical lens or microscope objectives, the spatial confinement of the light field is inevitably limited by diffraction. Specifically, when considering an object of radius *a*, the magnitude of the trapping force drops dramatically (following an ~*a*^3^ law) when the particle size is much smaller than the wavelength of the light [11]. To overcome the diffraction limitations, near-field optical manipulation techniques that use evanescent-type electromagnetic field configurations had to be introduced [12,13]. In this review, we focus our discussion on recent progress in the field of plasmonic optical tweezers (POT). In particular, we will discuss the opportunities available for researchers, current development bottlenecks and the limitations of the technology.

The POT field has two parent fields: optical tweezers and nanoplasmonics. The initial motivation was the existence of optical tweezers. Ashkin demonstrated that optical forces can levitate micron-scale particles in both water and air in the early 1970s [1,2]. A stable trap based on counterpropagating beams [2] eventually led to the development of the single-beam gradient force trap [3]. Ashkin and coworkers later used optical trapping in a wide range of experiments for the cooling and trapping of neutral atoms [4], viruses, bacteria and live cells [5,6]. The optical trap is created by focusing a laser beam to a diffraction limited spot, thus creating an optical potential for micron-scale objects. A focused laser beam can create an optical potential that is much larger than the thermal energy *k*_B_*T* required for stable trapping of a micron-sized particle. The field of optical tweezers has at least yielded fruitful results academically over the past four decades. In particular, single-molecule force spectroscopy has been enabled by the use of optical tweezers [10].

The second motivation was the development of nanoplasmonics and the closely-related subject of near-field optics [14,15]. The development of near-field optics stemmed from the realization that evanescent electromagnetic fields can overcome the diffraction limits of conventional far-field optics. The first near-field scanning optical microscope (NSOM) used a tapered optical fiber with a metalized nanoscale tip to generate an intensified light field with spatial features that were much smaller than the wavelength. In 1984, the IBM group presented the first optical images from this type of near-field optical microscope [16], and the technique was subsequently extended by Betzig *et al*. to multiple applications, such as the detection of single fluorescent molecules [17,18]. Plasmonics is based on the collective oscillation of electrons in metals. In a metallic nanostructure exhibiting plasmonic resonance, the resonant frequency depends on the restoring force, the effective electron mass and the dielectric constant of the metal [14,15]. Nanoplasmonics offers the capability of light confinement well below the optical wavelength because of the existence of optical modes that are localized based on dimensions that are much smaller than the optical wavelength. These modes are called surface plasmons, and they are essentially the eigenmodes of collective electronic oscillations. To enable a system to support surface plasmons, the dielectric permittivity of the material should contain both positive and negative components. A metallic material with negative dielectric permittivity supports the evanescent electromagnetic field, which decays in magnitude within the skin depth limit inside the material. A surface plasmon is characterized by the quality factor (*Q)*, which is defined in terms of the real and imaginary parts of the metal’s permittivity (ɛ_m_) as *Q* = −Re ɛ_m_/Im ɛ_m_, and is a measure of how many oscillations a surface plasmon will undergo before it loses its phases and energy. It can be shown that the local surface plasmon fields are enhanced by a factor of *Q* when under external excitation. For noble metals, such as gold, silver and aluminum, the maximum value of *Q* ranges from 10 up to 100.

## 2. Current Status

By combining the idea of nanoplasmonics and optical tweezers, Novotny, Bian and Xie proposed that a metalized tapered fiber illuminated using a laser beam can create sufficient optical potential to trap a nanoscale object in 1997 [19]. The near-field electromagnetic field can be greatly enhanced in a simple manner by “lightning rod” effects (where “lightning rod” effects refer to the fact that the electric field is strongest at the sharp tip of a lightning rod). The plasmonic oscillation further enhances the light intensity when the plasmon resonance conditions are met. Similar proposals have also been presented by other researchers [20,21]. The experimental realization of this proposal came almost a decade after the initial theoretical proposal based on plasmonics. On a thin gold film, Garcés-Chávez *et al.* [22] demonstrated the extended organization of 5-μm colloidal silica microparticles using a combination of thermal and optical forces. They observed a predominantly (hexagonal) close-packed crystalline arrangement of an array of approximately 2800 particles. Volpe *et al.* demonstrated the first transfer of momentum from a surface plasmon to a single dielectric sphere with a diameter of 4.5 μm, where the magnitude of the force at resonance is enhanced by 40-times in comparison to that under nonresonant illumination [23]. A series of similar experiments were subsequently reported [24,25]. Grigorenko’s group [26] reported the trapping of 200-nm particles using a conventional optical tweezers setup and scanning the nanoparticle across coupled pairs of gold nanodots and observed the quenching of Brownian motion by almost an order of magnitude. They also measured the trapping force by using a combination of the escape velocity and the modified Stokes law (the escape velocity *v*_esc_ is the speed at which the particle is unable to follow the laser beam of the optical tweezers when the nanoparticle is transported across the nanodot array). Quidant’s group reported the trapping of *E. coli*, representing the first trapping of bacteria using an optical antenna at 800 nm, which is a low photodamage wavelength [27]. The trapped *E. coli* bacteria continue to grow and divide in the nutrient medium for two hours. The optical antenna that was used for trapping consisted of two adjacent 500 nm-long gold wires with a 30-nm gap, and the rod-shaped *E. coli* bacteria also aligned well with the optical antenna’s long axis under the trapping conditions. Martin’s group reported the trapping of 10-nm metallic nanoparticles in the gap between dipole antennas [28]; this trapping process also caused a red shift in the plasmonic resonance and resulted in a change in the scattering intensity, which could then be used to monitor the trapping event. Similarly, self-induced back action (SIBA) has also been shown to enhance the force of POT [29]. SIBA is based on the concept that the presence of a dielectric particle can alter the plasmonic resonance of the metallic nanostructure and play an active and favorable role in enhancing the trapping force. Quidant’s group used a nano-aperture as a model system and demonstrated that the force calculated by the Maxwell stress tensor method in a non-perturbative manner can be greater than that calculated in a perturbative manner with the dipole approximation.

**Figure 1:**
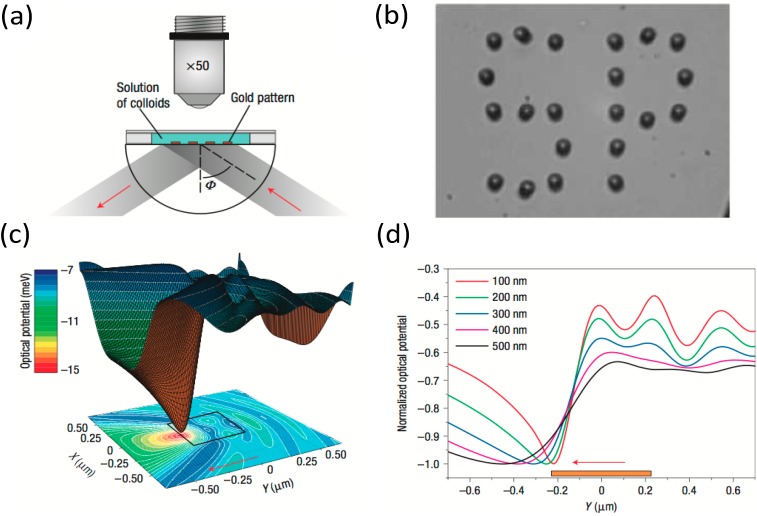
Plasmonic optical tweezers (POT). (**a**) Schematic diagram of typical optical configuration with prism coupling; (**b**) Arrangement of polystyrene microbeads on a patterned substrate after laser illumination at the surface plasmon resonance; (**c**) Computed optical potential landscape for a 200-nm polystyrene bead near a 0.45-μm-sided square gold pad; (**d**) Cross-sections of optical potential energy along the *Y* direction computed for different bead sizes. Reproduced with permission from [16]. Copyright 2008, Nature Publishing Group.

At present, a typical POT experiment requires a series of generic components: a light source for excitation of the plasmonic resonance; nanostructures with the required optical design; optical readout of the position of the dielectric particle being trapped; and various other devices (e.g., microfluidic systems to introduce the sample). Figure 1 shows a representative example of the experimental POT configuration. Despite the simplicity of this experimental scheme, POT has become a powerful technique for trapping of nanoscale objects, and the number of research results appearing in the literature on POT has increased dramatically. The field has, to date, centered on the demonstration of concepts for these components. The excitation of the plasmonic resonance can be provided by a laser beam via different coupling methods, such as prisms [23,24,25] and microscope objectives [26]. The electromagnetic fields of nanostructures are generally designed with the assistance of the finite difference time domain (FDTD) method. The optical force induced by the electromagnetic field can be calculated using the expression:

(1) $$\langle \vec{F}\rangle =\int_{\partial V}\langle \overset{↔}{T}(\vec{r},t)\rangle \cdot \hat{n}da$$

where ∂*V* denotes the surface of a volume enclosing the irradiated structure and $\overset{↔}{T}$ is the Maxwell stress tensor [14]. For the particle size *a*, which is much smaller than the wavelength and the characteristic length of the electromagnetic field distribution, the conditions for Raleigh scattering are satisfied, and the optical forces can be calculated by treating the particle as a point dipole [9]. The optical forces can then be separated into scattering and gradient forces, and in the application to POT, the optical gradient force is dominant and is given by the expression:

(2) $${\vec{F}}_{grad}=\frac{2\text{πα}}{cn_{m}^{2}}\nabla I_{0}$$

where *I*_0_ is the intensity distribution of the electromagnetic field and α is the polarizability of the particle being trapped and is given by:

(3) $$\text{α}=n_{m}^{2}a^{3}\frac{{\left(n_{p}/n_{m}\right)}^{2}-1}{{\left(n_{p}/n_{m}\right)}^{2}+2}$$

*n_p_* and *n_m_* denote the refractive indices of the particle and the surrounding medium, respectively. Various nanostructured designs, including nanodiscs [16], nanoholes [29] and coupled dipole antennas [27], have been used for POT. For isolated nanodiscs, one way to obtain the plasmon resonance is to treat these nanodiscs as prolate spheroids and apply the quasi-static approximation [14]. Nanoholes can also support plasmonic resonance, and optical transmission through the nanoholes can thus be used as the sensing mechanism [28,29]. Conceptually, it is very useful to treat these plasmonic nanostructures as nanoantennas [30]. The function of an antenna is based on the charge distribution being restricted to a well-defined region in space, and oscillation of such a charge distribution can produce both the far-field and the near-field. This perspective offers an intuitive insight that is especially useful for coupled nanostructures, such as coupled dipole antennas.

The POT nanostructures can be fabricated from noble metals using various top-down and bottom-up fabrication techniques [30]. The top-down techniques include electron beam lithography and focused ion beam milling (FIB). In a typical electron beam lithography method using the lift-off technique, the electron resist is patterned with a focused electron beam, developed and then selectively removed. A layer of metal can then be deposited, and a solvent is used to dissolve and remove the electron resist. In a typical FIB technique, the metal is deposited first, by methods such as thermal or electron beam evaporation. The metallic layer is then patterned, using a focused ion beam to remove the unwanted region. The main advantage of this process is that the FIB technique offers very good resolution and also allows the patterning of chemically-grown single crystalline metal flakes [31]. The bottom-up approaches are based on either chemical synthesis or self-assembly of nanoparticles in solution. The main advantages of these approaches are that near-perfect symmetry and crystallinity can be achieved over large substrate areas and with low fabrication costs. In terms of material choices, gold is the prevalent material selection for POT nanostructures, because it provides ease of fabrication and is chemically inert [5]. In comparison, silver is known to corrode rapidly, and aluminum is known to form thin aluminum oxide passivation layers. The spectral properties of gold also make it an excellent material to produce the maximal *Q* of ~10 in the near infrared wavelength region. This is particularly important for biological applications, because the photodamage caused to live cells is minimal in the near infrared region.

The optical readout can be simple direct optical imaging by a camera [23,24,25] or optical transmission [29]. The recorded image can be post-processed using an algorithm to extract the particle trajectory and velocity data. The optical transmission data yield the trapping event kinetics. Nanoparticle trapping is mostly carried out in liquids, and the nanoparticle sample is often placed in a fluidic cell to prevent evaporation. For lab-on-a-chip applications, a microfluidic device, such as a polydimethylsiloxane (PDMS) chip, can also be integrated into the device to deliver the dielectric particle appropriately for trapping [32]. In particular, multilayer soft lithography of PDMS can be used to fabricate devices, such as microvalves, to enable active plumbing. This type of integration will greatly expand the potential for usage of POT in practical applications.

## 3. Future Prospects

### 3.1. Molecular Manipulation

**Figure 2:**
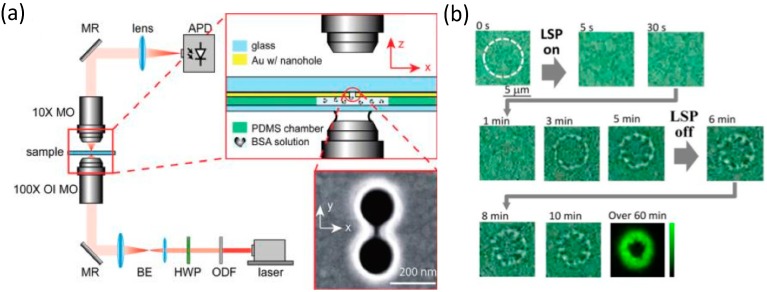
Molecular manipulation with plasmonic optical tweezers. (**a**) Schematic of experimental setup for trapping of a single bovine serum albumin (BSA) protein. The single BSA protein is trapped in the gap of a double-hole nanostructure. The nanostructure is shown in the inset scanning electron microscope (SEM) image. Reproduced with permission from [33]. Copyright 2012, American Society of Chemistry. (**b**) Micro-pattern of DNA on a substrate patterned with a gold nanostructure. Reproduced with permission from [37]. Copyright 2013, American Society of Chemistry.

While the field of optical tweezers has produced a large database for single-molecule force spectroscopy, the experimental protocol often relies on tethering of a single molecule to a micron-sized bead as a handle. Direct manipulation of a single molecule, such as a protein, and interrogation of its molecular configuration is still at the top of many experimentalists’ wish lists. As shown in Figure 2a, Gordon’s group has demonstrated trapping of a single bovine serum albumin (BSA) protein molecule in a double-hole nanostructure [33]. Using optical transmission, they were able to observe the transition between two protein folding configurations. Different BSA folding configurations are manifested as different optical transmission levels. Using the same technique, Gordon’s group has also shown that a double-nanohole optical trap can distinguish the bound and unbound forms of a single protein, the binding on zipping and unzipping of single DNA hairpins and the single-molecule protein binding kinetics of a single human serum albumin (HSA) [34,35,36]. Tsuboi’s group has reported permanent fixing and POT trapping of λ-DNA molecules stained with YOYO-1 fluorescent dye with cw and femtosecond lasers [37], as shown in Figure 2b. For the plasmonic nanostructures, they fabricated gold nanopyramidal dimer arrays on a glass substrate by means of nanosphere lithography, and the cw and femtosecond lasers for excitation of plasmonic resonance were loosely focused to a circular spot of ~5 μm in diameter. In Figure 2b, the time shown is the cw laser turn-on time, and the white dashed circle indicates the region of cw laser illumination. His group also reported POT trapping of polymer chains [38]. The advantage of POT for molecular manipulation is that the method can be combined with various spectroscopy techniques, such as surface-enhanced Raman spectroscopy, to determine the details of the molecules of interest.

### 3.2. Rotation by Plasmonic Optical Force and Torque

In addition to transfer of linear momentum to generate a radiation force, POT can also impart angular momentum to nanoscale objects to induce rotational movement. The conservation of angular momentum of the electromagnetic field can be expressed mathematically as:

(4) $$-\int_{\partial V}\langle \overset{↔}{T}(\vec{r},t)\times \vec{r}\rangle \cdot \hat{n}da=\frac{d}{dt}(J_{field}+J_{mech})$$

where $\overset{↔}{T}$, ${\vec{J}}_{field}$ and ${\vec{J}}_{mech}$ are the Maxwell stress tensor, the electromagnetic angular momentum and the mechanical angular momentum [14]. ∂*V* denotes the surface of a volume that encloses the irradiated structure. The angular momentum that is carried by the photons can induce a mechanical torque, which can be calculated for a monochromatic field as:

(5) $$\langle \vec{N}\rangle =-\int_{\partial V}\langle \overset{↔}{T}(\vec{r},t)\times \vec{r}\rangle \cdot \hat{n}da$$

**Figure 3:**
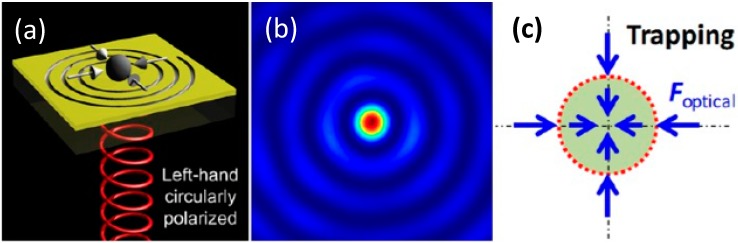

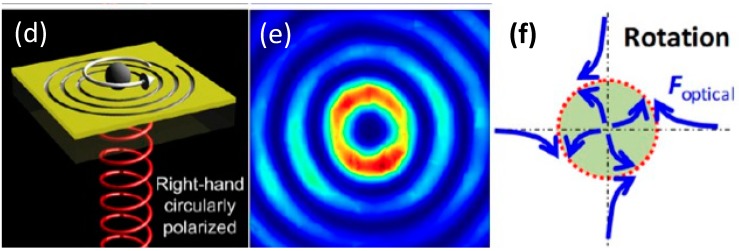
Plasmonics induced rotation of microparticles. (**a**) Under left-hand circularly-polarized plane wave excitation, the EM field is focusing with no angular momentum to trap the microparticles; (**b**) The intensity distribution of (a) is shown; (**c**) Schematic of the trapping force; (**d**) Under right-hand circularly-polarized plane wave excitation, the EM field imparts angular momentum to rotate the microparticles; (**e**) The intensity distribution of (d) is shown; (**f**) Schematics of the trapping forces. Reproduced with permission from [37]. Copyright 2013, American Society of Chemistry.

The transfer of angular momentum from an optical beam to an irradiated object was first measured by Beth [39]. An optical beam with non-zero angular momentum can also cause a trapped particle to spin, and various applications of this phenomenon have been suggested [40,41]. For example, spinning micro-objects can serve as microblenders for localized mixing in microfluidic systems. Using plasmonics, Crozier’s group changed the polarization to rotate a nanoparticle along the perimeter of a gold nanodisc with an appropriate design for heat sinking [42]. As shown in Figure 3, Huang’s group (one of the authors) has used an Archimedes spiral structure to generate an optical vortex and to induce orbital rotation in a linear assembly of microparticles [43]. Depending on the chirality of the input circularly-polarized light, the microparticles showed selective particle trapping directed towards the spiral origin by a focusing spot or particle rotation along the primary ring caused by a plasmonic vortex field. To date, the self-spinning of a nanoparticle induced by a plasmonic device has not yet been demonstrated. A related work by Zhang’s group has also shown that a silica microdisc with an embedded plasmonic nanostructure can be driven to rotation by light [44].

### 3.3. Plasmon-Assisted Optofluidics with POT

The metallic plasmonic nanostructure used in POT can act as a point-like heat source, and this effect often complicates the situation in practice by inducing convective fluidic flow. Garcés-Chávez *et al.* [22] have observed fluid flow induced by both thermophoresis and by convection due to heating on a gold thin film. Thermophoresis occurs with increasing optical power and results in the repulsion of particles and a ring-shaped particle aggregation pattern. If the convection flow caused by photothermal effects is properly controlled, it can also be used to trap and manipulate micro- and nano-particles. Quidant’s group has used a Green’s function approach to calculate the temperatures of plasmonic nanostructures of various shapes, such as nanospheres and nanodiscs [45,46]. The electrical field is obtained using the discrete dipole approximation, and the steady-state temperature profile is the solution of the Poisson equation:

(6) $$\text{κ}\nabla^{2}T(\vec{r})=-q(\vec{r})$$

where κ is the thermal conductivity of the medium and $q(\vec{r})$ is the heat source density. In the presence of the substrate, Green’s function, which is defined as the temperature profile of a point heat source, can be obtained using the image method that is usually used in electrostatics. Because the thermal conductivity of the metallic nanostructures is much greater than that of the substrate materials, such as glass or indium tin oxide (ITO), the temperature distribution is uniform across the metallic nanostructures [46]. The same group later gave analytical expressions for the temperature rise in arrays of nanoparticles under uniform or Gaussian beams and experimentally validated these expressions using measured temperature profiles [47]. For a given temperature profile, the fluid dynamics are governed by the steady-state Navier–Stokes equation with the Boussinesq approximation at low Reynolds numbers [48]:

(7) $$\text{η}\nabla^{2}\vec{v}(\vec{r})+{\text{ρ}}_{fluid}{\text{α}}_{fluid}\vec{g}[T(\vec{r})-T_{0}]=0$$

where η, ρ_*fluid*_, α_*fluid*_ and *T*_0_ are the dynamic viscosity, the fluid density, the thermal expansion coefficient and the reference temperature of the fluid, respectively. $\vec{v}$ and $\vec{g}$ are the fluid velocity profile and gravitational acceleration. This theoretical investigation of the temperature profile by Quidant’s group has led to an analytical expression for the order of magnitude of the velocity:

(8) $$V\approx L^{2}{\text{ρ}}_{fluid}{\text{α}}_{fluid}gT_{0}/\text{η}$$

for the typical length scale *L* [49]. The estimates from this analytical formula are also consistent with the results of numerical calculations, which show that the convective velocity remains negligible (*i.e*., <1 nm/s) for isolated nanoplasmonic structures. Toussaint’s group performed calculations of the convective flow from an array of nanoantennas on an ITO sample and concluded that the flow had high fluidic velocity of the order of micrometers per second [50]. The calculations show that the optically-absorptive and highly thermally-conductive ITO can distribute the thermal energy more efficiently and increase the convective velocity by an order of magnitude when compared with a nanoantenna on a less thermally-conductive SiO_2_ substrate layer. This work opened new avenues for the control of fluid and mass transport on micro- and nano-scale plasmonic structures. Convective fluidic flow can act as an auxiliary mechanism to bring a nanoparticle over a comparatively long distance into the minute trapping volume of the POT. Toussaint’s group also established a phase diagram for a bowtie antenna array for single and multiple particle stacking and sorting as a function of input power, wavelength, polarization, particle diameter and array spacing [51]. The optically- and thermally-induced forces were tuned to generate the following three distinct states of trapping: (1) lateral delocalization; (2) single-particle trapping; and (3) multiple trapping of two-dimensional (2D) and three-dimensional (3D) hexagonally-packed clusters. The photothermal heating of the plasmonic nanostructure array can also be combined with an AC electrical field of ~100 kHz to enable electrokinetic manipulation [52]. This combination creates a strong electrothermal fluid flow with velocities as high as 50 μm/s. Sorting of particles of 1 μm and 2 μm in size can also be demonstrated by switching the frequency of the AC electrical field. The plasmonic nanostructure shows a much stronger flow velocity than that of the thin gold film substrate because of the near-resonance excitation.

### 3.4. POT with Plasmonic Optical Lattice

**Figure 4:**
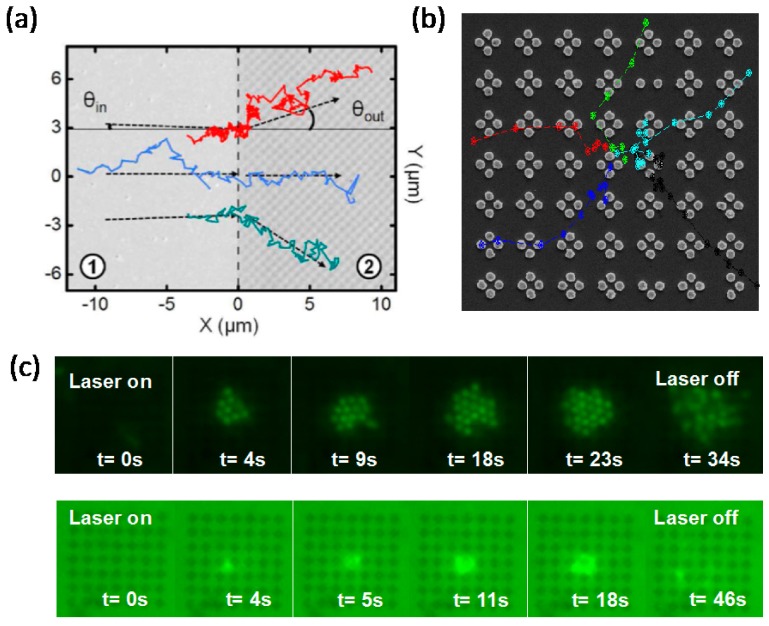
Trapping and transport in plasmonic optical lattices. (**a**) Real space trajectory showing the negative refraction of a nanoparticle crossing the interface between Region 1 (a flat gold thin film) and Region 2 (an undulating gold thin film). Reproduced with permission from [57]. Copyright 2012, American Chemistry Society. (**b**) Single-particle trajectory over a nanoscale two-dimensional plasmonic optical lattice; (**c**) Multiple particle stacking over the same plasmonic optical lattice that is shown in (b). Reproduced with permission from [58]. Copyright 2013, American Chemistry Society.

Another frontier in this field is the plasmonics enhanced optical lattice. The statistical transport of micro- and nano-particles under a periodic potential established by conventional optical tweezers has many interesting applications. Optical fractionation based on kinetic lock-in is one example [53,54]. When particles are forced across a periodic potential, they can either follow the driving force or a specific trail in that periodic potential. The second choice is called kinetic lock-in and is only apparent when the directions of the force and the trail are aligned at a slight angle to each other. Particles of different sizes or with different refractive indexes can follow different directions (either locked or unlocked), even when they are originally introduced as a mixture. Such a phenomenon can then be used to perform sorting based on particle size or refractive index in microfluidic environments [55]. It would therefore be interesting to scale these experiments down to the nanoscale using nanoplasmonics. However, when the instrumentation complexity and high optical power requirements of optical lattices formed by holographic or interference techniques are considered, optical lattices for POT applications can be realized in a simpler form. Cuche and coworkers have reported the first transport experiment over a plasmonic optical lattice [56,57]. As shown in Figure 4a, they demonstrated negative refraction phenomena in nanoparticle transport when the nanoparticles roam across the interface between a flat gold film and an undulating gold film. The authors’ group has also reported on trapping and transport over a periodic array of gold nanodiscs [58]. The array was illuminated by a loosely-focused expanded Gaussian beam, and both single and multiple particle trapping were reported, as shown in Figure 4b,c. This conceptual advance from single, isolated POT to arrays of POT offers long-range transport for nanoparticles and greatly extends the usefulness of this technology.

### 3.5. New Nanobiotechnology and Lab-on-a-Chip Applications

The ability to trap nanoscale objects with low optical power makes POT an ideal tool for trapping of biological objects, such as viruses and vesicles. Viruses are highly important biological entities that affect many aspects of human life and are potentially the killer application for POT [59]. The entire repertoire of known viruses ranges in size from micrometers to nanometers and, thus, fits well within the trapping range of POT. The infectious prion, which is loosely defined as the smallest virus, is a few nanometers in size. While viruses can be captured by binding with antibodies, POT offers the advantage of capturing and releasing viruses by simply switching light on and off. Furthermore, the use of POT means that no specific antibodies are required for the process, and a gold POT nanostructure is considerably more stable than an antibody. Extracellular vesicles (EVs), which are membranous particles that are released by various types of cells, hold great potential for use in clinical applications [60]. They contain nucleic acid and protein cargos and are increasingly recognized as a means of intercellular communication used by both eukaryotic and prokaryotic cells. The involvement of EVs in a variety of cellular processes, including intercellular communication, immunity modulation, angiogenesis, metastasis, chemoresistance and the development of eye and neural diseases, is being increasingly recognized and has spurred great interest in the use of EVs for diagnostic, prognostic, therapeutic and basic biological applications. However, because of their heterogeneity and small size (40 nm to 5000 nm in diameter), current protocols for EV detection often involve a series of ultracentrifugation and/or ultracentrifugation steps followed by molecular analyses, which are time consuming and cumbersome processes that require large sample volumes. The integration of a lab-on-a-chip device with POT will enable the study of EVs and, particularly, of their utility as reliable predictors of altered physiology or the development of diseases. While these two examples are important potential applications, there are no reports of the trapping of viruses or vesicles using POT in the literature to date.

For lab-on-a-chip applications, there are at least three optical characteristics of POT that make these devices highly attractive for integration with microfluidics systems: low excitation optical power, low *Q* and no need for a high numerical aperture objective. For gold, the most prevalent material for POT, the bulk *Q* value is ~10, and the plasmonic resonance is therefore sufficiently broad to ensure that no tunable laser source is needed. In fact, the excitation source does not need to be coherent, and thus, an incoherent source, such as a light-emitting diode, will work just as well for trapping purposes, because the typical time scale required for trapping is usually much longer than the coherence time. For comparison, a silicon photonic crystal waveguide that was used for trapping has a very high *Q* of the order of ~1000 and would need an expensive and cumbersome tunable laser for the trapping operation [61]. This would greatly impede its usability for all optical lab-on-a-chip applications.

### 3.6. Atomic Physics

**Figure 5:**
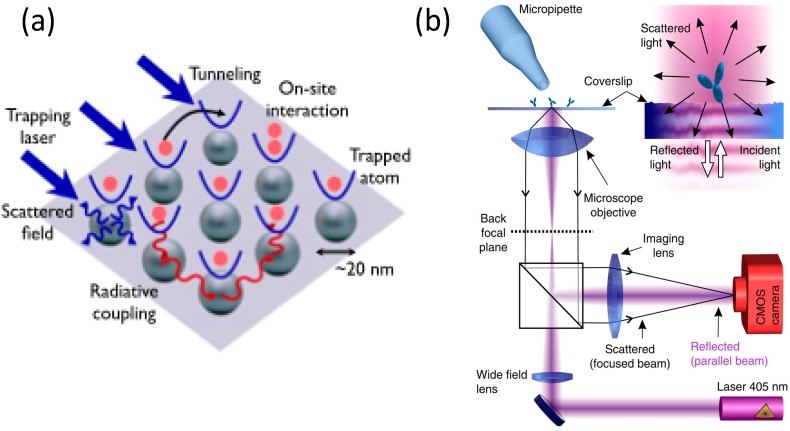
Future opportunities for POT. (**a**) Physical processes involved in atomic trapping in a nanoplasmonic optical lattice. Reproduced with permission from [63]. Copyright 2012, American Physical Society. (**b**) Interferometric scattering microscopy (i-SCAT) for label-free detection of single molecules. Reproduced with permission from [65]. Copyright 2012, Nature Publishing Group.

The first theoretical proposal for cooling of atoms in counterpropagating laser beams was made by Hänsch and Schawlow in 1975 [62]. Chu and Ashkin first demonstrated the optical trapping of atoms [4], and this work was the starting point for a series of experiments that eventually led to the 1997 Nobel Prize in Physics. In short, the mechanical force involved in laser trapping and cooling of atoms can be understood on a semiclassical basis by treating the atom as a quantized two-level system, but the quantum theory of photons must be used for correct interpretation of the results [63]. POT can also open a new avenue for atomic trapping. In particular, the trapping of atoms in a plasmonic optical lattice will enable the study of quantum mechanical interactions between atoms. Considerable interest has been directed towards the development of an atomic system using plasmonic nanostructures to trap and coherently manipulate atoms within a range up to ~100 nm above the surface. Chang *et al.* have theoretically proposed the plasmonic trapping of single atoms [64]. They analyzed a scheme for interfacing of individual neutral ^87^Rb atoms with a silver nanotip. The guided surface plasmon modes allow the atom to be manipulated optically or allow fluorescence photons to be collected with very high efficiency. Gullen *et al.* later extended this proposal to consider atomic trapping in nanoplasmonic optical lattices and concluded that pushing of the spacing in the nanoplasmonic optical lattice can significantly increase the coupling and, thus, enable the observation of many-body effects [65]. They considered an array of metallic nanospheres in a vacuum illuminated by a plane wave and used the self-consistent dipole approximation to calculate the field and, thus, obtained the trapping potentials. Their calculations showed that both on-site and tunneling interactions for the Hubbard model can be increased by several orders of magnitude when compared with the optical lattice, as highlighted in Figure 5a. To date, these proposals have not been demonstrated experimentally. However, the trapping of single atoms using a one-dimensional silicon photonic crystal waveguide has been demonstrated by Lukin’s research group [66].

### 3.7. Combination with Other High-Resolution Microscopy

POT technology can manipulate objects of interest with nanometer resolution, and position readout with matching precision is required to realize the full potential of POT. This is currently performed using direct (fluorescent or non-fluorescent) optical imaging and optical transmission techniques. Optical transmission methods can detect the presence of the object being trapped and yield information, such as the trapping kinetics. However, the information that can be provided is very limited when compared with that provided by direct optical imaging methods. Direct optical imaging can provide information, such as single and/or multiple particle trajectories and the spatial arrangement of the particle assembly. At present, for objects that are smaller than the wavelength of the light, fluorescent imaging is still the prevalent technique. The electron-multiplying charge-coupled device (EMCCD) camera can easily provide the sensitivity required for single-molecule detection. However, the fluorescent technique is limited, because not all molecules or objects are suitable for fluorescent labeling, and photobleaching will limit the number of photons available for detection and, thus, will also limit the maximal attainable signal-to-noise ratio. The combination of POT with another label-free, single-molecule resolution, the high temporal and spatial resolution microscopy technique, such as interferometric scattering microscopy (i-SCAT) [67], therefore becomes a very attractive prospect. The i-SCAT technique detects the scattering signal and uses a homodyne measurement method based on the interference of the detected signal with a local oscillator, as shown in Figure 5b. This detection method pushes the noise performance into the shot noise-limited regime and is not limited by photobleaching. i-SCAT is thus considered to be a very attractive candidate auxiliary imaging technique for POT.

## 4. Conclusions

The use of POT extends the particle trapping range down to the nanometer scale and, therefore, opens up unprecedented opportunities for application in many scientific fields. POT can be integrated with a lab-on-a-chip to provide a promising technique for capture and detection of nanoscale biomaterials, such as viruses and vesicles. Direct manipulation of single molecules will enable label-free and untethered trapping and will greatly extend the range of molecules that can be interrogated. On the one hand, it seems that the field of POT has a clear roadmap to follow that has been defined by conventional optical tweezers. Like the optical lattice formed by conventional optical tweezers, plasmonic optical lattices will provide a platform for the study of nanoscale transport of nano-objects in liquid environments. Milestones, such as trapping of atoms with POT, now need to be demonstrated. POT can be extended to trapping and manipulation of individual atoms to explore their coherent interactions and quantum many-body effects. On the other hand, there are also issues that are unique to POT. For example, the photothermal effect should be minimized in general via optimal heat sinking, because it tends to interfere with trapping, and high temperatures will also damage biological specimens. A temperature measurement capability with high spatial resolution and simple implementation should therefore be a standard part of the POT apparatus.

The physics of POT will continue to be explored in its own right. For example, a recent theoretical proposal addressed a plasmonic thermal ratchet with asymmetric optical potential for turning the illumination on and off [68]. However, the question of whether or not this can be realized experimentally remains open. Although nanostructure design for POT largely relies on computer simulations, theoretical formalisms that yield analytical formulas are equally necessary to guide the experimental design in a more intuitive manner. For example, it should be possible to formulate the SIBA effect in a more rigorous way by combining the resonance shift theory with force calculations [69]. A theoretical formulation for electrokinetic manipulation using plasmonic nanostructures is also required to take the contributions from thermal effects, optical gradients and AC electrical field enhancement into account. Finally, as the field matures, POT instruments should no longer be confined to laboratories that build their own custom setup. Change should be driven by increased availability of commercial systems, perhaps in the lab-on-a-chip format. POT can be a standalone system that manipulates and analyzes nanoparticles of interest by using low-cost and disposable metallic nanostructures on a chip. Alternatively, POT technology can be available as a component of the current commercial systems, such as near-field scanning optical microscopes (NSOM).
